# Gas as a Health Preserver

**Published:** 1886-06

**Authors:** 


### GAS AS A HEALTH PRESERVER.

It has been known almost since the establishment of gas works that the exhalations arising in the various processes of gas manufacture, although, perhaps, not specially pleasing to the olfactory organs, are not detrimental to health, but are, on the contrary, highly beneficial in special forms of disease, such as whooping cough and croup. The extensive use, in throat ailments, of preparations in which some form of carbolic acid figures largely, is a testimony to the value of this derivative of coal tar as a therapeutic agent. A recent issue of the *Journal des Usines a Gaz* contained particulars respecting certain investigations, made by Dr. Lemaire some years ago, into the subject of the influence of coal tar and its derivatives upon the health of the workmen employed in the preparation of these substances. His inquiries were made chiefly in connection with the employes of the Paris Gas Company. He found that those whose duties did not necessitate a prolonged stay in the parts of the works where tar was to be found were liable to all kinds of ailments, and formed a considerable proportion of the number of the sick list, while among the workmen specially occupied with tar, only three were sick in the course of seven years. This result is all the more striking when the number of workmen in the service of the company at the period referred to is considered. There were altogether 20,553 men, of whom 764 were engaged in some occupation connected with tar. Dr. Lemaire also cites the case of the Bayonne Gas Works, where the workmen had not only not been attacked by cholera during its prevalence, but generally enjoyed immunity from skin diseases. M. Bouley, a professor at the Veterinary School, at Alford, found, as long ago as 1860, that gas-work employes escaped during cholera epidemics, and the communication of this fact to Dr. Lemaire caused him to institute his inquiries into the subject.—*Scientific American.*

